# Optimization of the Sampling Periods and the Quantization Bit Lengths for Networked Estimation

**DOI:** 10.3390/s100706406

**Published:** 2010-06-29

**Authors:** Young Soo Suh, Young Sik Ro, Hee Jun Kang

**Affiliations:** Department of Electrical Engineering, University of Ulsan, Namgu, Ulsan 680-749, Korea; E-Mails: ysro@ulsan.ac.kr (Y.S.R.); hjkang@ulsan.ac.kr (H.J.K.)

**Keywords:** networked estimation, sampling periods, quantization, Kalman filter

## Abstract

This paper is concerned with networked estimation, where sensor data are transmitted over a network of limited transmission rate. The transmission rate depends on the sampling periods and the quantization bit lengths. To investigate how the sampling periods and the quantization bit lengths affect the estimation performance, an equation to compute the estimation performance is provided. An algorithm is proposed to find sampling periods and quantization bit lengths combination, which gives good estimation performance while satisfying the transmission rate constraint. Through the numerical example, the proposed algorithm is verified.

## 1. Introduction

Recently, networked monitoring systems are becoming increasingly popular, where sensor data are transmitted to a monitoring station through wired or wireless networks [1, 2]. In a monitoring station, estimation algorithms (such as a Kalman filter) are used to estimate the system states. A network between sensor nodes and a monitoring station can induce many problems such as time delays, packet dropouts, and limited bandwidth, where they depend on network types and scheduling methods. We note that the network issue (for example, what kinds of scheduling methods should be used?) itself is a big research area [3]. Also time delays and packet dropouts [4–7] are one of most important problems in networked estimation problems.

In this paper we focus on the case where there are many sensors and the network bandwidth is limited. For example, suppose there are three sensors (A,B,C) and 90 bytes/s can be transmitted over a network. How does one assign 90 bytes/s to each sensor? One method is to assign 30 bytes/s to each sensor. If sensor A monitors fast changing value and sensor B monitors slowly changing value, it might not be the best strategy: more data rate should be assigned to sensor A and the data rate of sensor B should be reduced. These issues are discussed quantitatively in this paper.

Note that the data rate of each sensor depends on the sampling frequency and the quantization bit length. For example, (100 Hz, 8 bit) case and (50 Hz, 16 bit) case have the same data rate (100 bytes/s). Thus given the same data rate, we have many possible combinations of the sampling frequencies and the quantization bit lengths. We first investigate how the sampling frequency and the quantization bit length affect the estimation performance and then propose a method to choose the sampling frequency and the quantization bit length of each sensor.

Using different sampling frequencies for different sensors is discussed in [10], where the sampling frequencies is chosen by minimizing the Kalman filter error covariance. In [11], the sampling frequency assignment algorithm is given, where a sampling frequency is chosen from a finite discrete set. In [12], a similar sampling frequency assignment is considered, where location of sensors and cost of measurement are also considered in the optimization problem. We note there are other attempts, where an event-based transmission method [8, 9] is used instead of a periodic transmission. In this paper, we assume that periodic sampling of sensor data.

Quantization is an extensively studied area [13]. Relating the estimation problem, a logarithm quantizer is proposed in [14]. Although theoretically appealing, the quantizer is applied to the innovation of a filter rather than to an output directly. The effect of quantization can be reduced by treating the quantization error as measurement noises as in [15]. In [16] and [17], quantization bit length assignment algorithms are proposed, where the bit length is computed by minimizing a performance index. The performance index is not directly related to estimation performance (e.g., the filter error covariance).

Simultaneous optimization of the sampling frequency and the quantization bit length has not been reported yet. In this paper, both parameters are selected so that the estimation performance is optimized given the transmission rate constraint.

The paper is organized as follows. In Section 2., estimation performance *P* is defined, which depends on the sampling periods and quantization bit lengths. In Section 3., a suboptimal algorithm to compute sampling period and quantization bit length combination is proposed. Through numerical examples, the proposed method is verified in Section 4. and conclusion is given in Section 5.

## 2. Problem Formulation

In this section, estimation performance is defined when the sampling frequency and the quantization bit length of each sensor are given. How to optimize the sampling frequency and the quantization bit length is discussed in Section 3.

Consider a linear time-invariant system given by

(1) $$\begin{array}{lll} \dot{x}(t) & = & Ax(t)\;+w(t)\\ y(t) & = & Cx(t)\;+v(t) \end{array}$$

where *x* ∈ *R^n^* is the state we want to estimate and *y* ∈ *R^p^* is the measurement. Process noise *w*(*t*) and measurement noise *υ*(*t*), which are uncorrelated, zero mean white Gaussian random processes, satisfy:

$$\begin{array}{lll} E\{w(t)w(s)\prime \} & = & Q\delta (t-s)\\ E\{v(t)v(s)\prime \} & = & R\delta (t-s)\\ E\{w(t)v(s)\prime \} & = & 0 \end{array}$$

where E{·} denotes the mean and *Q* and *R* are a process noise covariance and a measurement noise covariance, respectively.

Let *T_i_* be the sampling period of the *i*-th output and thus the corresponding sampling frequency is 1/*T_i_*. We assume that *T_i_* is an integer multiple of constant *T*: that is, *T_i_* satisfies the following condition:

(2) $$T_{i}\;=\;M_{i}T$$

where *M_i_* is an integer and *T* is the base sampling period.

Let *l_i_* be the quantization bit length of the *i*-th output. Let *y_max,i_* be the absolute maximum value of the *i*-th output: that is,

(3) $$|y_{k,i}|\;\leq \;y_{\max,i}$$

where *y_k,i_* is the *i*-th element of *y_k_*. The index *k* is used to denote the discrete time index. We assume that the uniform quantizer is used. Let *δ_i_* be the quantization level of the *i*-th output, which is given by

(4) $$\delta_{i}\;=\;\frac{y_{\max,i}}{2^{l_{i}-1}}$$

Let *q_k_* be the quantization error in *y_k_*, then the following is satisfied

(5) $$|q_{k,i}|\;\leq \;\frac{\delta_{i}}{2}$$

where *q_k,i_* is the *i*-th element of *q_k_*.

Now we are going to model (1) in the discrete time considering the sampling period *T_i_* and the quantization length bit *l_i_*. Assume *M_i_* = 1 (1 *≤ i ≤ p*) temporarily:

(6) $$\begin{array}{lll} x_{k+1} & = & \Phi x_{k}\;+\;w_{k}\\ y_{k} & = & Cx_{k}\;+\;v_{k}\;+\;q_{k} \end{array}$$

where *x_k_* ≜ *x*(*kT*), Φ ≜ exp(*AT*) and *y_k_* is the quantized output of *y*(*kT*). Process noise *w_k_* and *υ_k_* are uncorrelated and satisfy

(7) $$E\{w_{k}w_{k}^{\prime }\}\;=\;Q_{d},\;E\{v_{k}v_{k}^{\prime }\}\;=\;R$$

where

$$Q_{d}\;≜\;\int_{0}^{T}\text{exp}(\mathrm{Ar})Q\text{exp}(\mathrm{Ar})\prime \;\mathrm{dr}$$

We treat the quantization error as an additional measurement noise as in (6), which is also considered in [15]. The quantization error *q_k_* is assumed to be uncorrelated with *w_k_* and *υ_k_*. If the uniform distribution is assumed, the covariance is given by

(8) $$E\{q_{k}q_{k}^{\prime }\}\;=\;\Delta \;=\;\text{Diag}(\frac{\delta_{1}^{2}}{12},\;\cdots ,\;\frac{\delta_{p}^{2}}{12})$$

Now the temporary assumption (*M_i_* = 1) is removed. The second equation of (6) is no longer true and *y_k,i_* is available if *k* is an integer multiple of *M_i_*. Let *ỹ_k_* be a collection of all available *y_k,i_* at time *k*. To define *ỹ_k_* in a more formal way, let {*r_k_*_,1_, *r_k_*_,2_, . . ., *r_k,p_k__*} be a set of all row numbers of available *y_k_*. Then *ỹ_k_* is given by

(9) $${\tilde{y}}_{k}\;≜\;\left[\begin{array}{c} \underline{y_{k,r_{k},1}}\\ \underline{y_{k,r_{k,2}}}\\ \underline{\cdots }\\ y_{k,r_{k,p_{k}}} \end{array}\right]$$

Similarly *υ̃_k_* and *q̃_k_* can be defined and *C̃_k_* is defined as follows:

$${\tilde{C}}_{k}\;=\;\left[\begin{array}{c} \underline{C_{r_{k,1}}}\\ \underline{C_{r_{k,2}}}\\ \underline{\cdots }\\ C_{r_{k,p_{k}}} \end{array}\right]$$

where *C_i_* is the *i*-th row of *C*. Thus the measurement equation at time *k* is given by

(10) $${\tilde{y}}_{k}\;=\;{\tilde{C}}_{k}x_{k}\;+\;{\tilde{v}}_{k}\;+\;{\tilde{q}}_{k}$$

where

(11) $$\begin{array}{lll} E\{{\tilde{v}}_{k}{\tilde{v}}_{k}^{\prime }\} & = & {\tilde{R}}_{k}\\ E\{{\tilde{q}}_{k}{\tilde{q}}_{k}^{\prime }\} & = & {\tilde{\Delta }}_{k} \end{array}$$

*R̃_k_* ∈ *R^p_k_×p_k_^* is a matrix extracted from *R* so that *R̃_k_*(*i*, *j*) = *R*(*r_k,i_*, *r_k,j_*). Δ̃*_k_* is defined in the same way.

For example, if *M*_1_ = 1 and *M*_2_ = 2, then *ỹ_k_* and *C̃_k_* are given in Table 1. We can see that *C̃_k_* is periodic with the period 2, which is the least common multiple of *M*_1_ = 1 and *M*_2_ = 2.

Generally *C̃_k_* is periodic with the period *M*, where *M* is the least common multiple of {*M*_1_, *M*_2_, . . ., *M_p_*}.

Using the first equation of (6) repeatedly *b* times, we have

(12) $$x_{b+a}\;=\;\Phi^{b}x_{a}\;+\;\sum_{i=0}^{b-1}\Phi^{b-1-i}w_{a+i}$$

It is known that a periodic system can be transformed into a time-invariant system [18, 19]. From (12) with *a* = *kM* and *b* = *M*, we have

(13) $$x_{(k+1)M}\;=\;\Phi^{M}\;x_{\mathrm{kM}}\;+\;\sum_{i=0}^{M-1}\Phi^{M-1-i}w_{Mk+i}$$

Also from (12) with *a* = *kM − j* and *b* = *j*, we have

(14) $$x_{\mathrm{kM}}\;=\;\Phi^{j}\;x_{\mathrm{kM}-j}\;+\;\sum_{i=0}^{j-1}\Phi^{j-1-i}\;w_{\mathrm{kM}-j+i}$$

Multiplying Φ^−^*^j^*, we obtain a backward equation:

(15) $$x_{\mathrm{kM}-j}\;=\;\Phi^{-j}\;x_{\mathrm{kM}}\;-\;\sum_{i=0}^{j-1}\Phi^{-1-i}w_{\mathrm{kM}-j+i}$$

Let *x̄_k_*, *w̄_k_* *ȳ_k_*, *ῡ_k_* and *q̄_k_* be defined by

$${\bar{x}}_{k}\;≜\;x_{\mathrm{kM}}$$

$$\begin{array}{cc} {\bar{w}}_{k}\;≜\;\left[\begin{array}{c} w_{\mathrm{kM}}\\ w_{\mathrm{kM}+1}\\ \vdots \\ w_{\mathrm{kM}+M-1} \end{array}\right], & {\bar{y}}_{k}\;≜\;\left[\begin{array}{c} {\tilde{y}}_{(k-1)M+1}\\ {\tilde{y}}_{(k-1)M+2}\\ \vdots \\ {\tilde{y}}_{kM} \end{array}\right]\\ {\tilde{v}}_{k}\;≜\;\left[\begin{array}{c} {\tilde{v}}_{(k-1)M+1}\\ {\tilde{v}}_{(k-1)M+2}\\ \vdots \\ {\tilde{v}}_{kM} \end{array}\right], & {\bar{q}}_{k}\;≜\;\left[\begin{array}{c} {\tilde{q}}_{(k-1)M+1}\\ {\tilde{q}}_{(k-1)M+2}\\ \vdots \\ {\tilde{q}}_{kM} \end{array}\right] \end{array}$$

Combining (13), (15) and (10), we have the following time invariant system:

(16) $$\begin{array}{lll} {\bar{x}}_{k+1} & = & \bar{A}{\bar{x}}_{k}\;+\;\bar{B}{\bar{w}}_{k}\\ {\bar{y}}_{k} & = & \bar{C}{\bar{x}}_{k}\;+\;{\bar{v}}_{k}\;+\;{\bar{q}}_{k}\;+\;\bar{D}{\bar{w}}_{k-1} \end{array}$$

where

$$\bar{A}\;=\;\Phi^{M}$$

$$\bar{B}\;≜\;\;\left[\Phi^{M-1}\;\Phi^{M-2}\;\cdots \;I\right]$$

$$\bar{C}\;≜\;\left[\begin{array}{c} {\tilde{C}}_{1}\Phi^{-M+1}\\ {\tilde{C}}_{2}\Phi^{-M+2}\\ \vdots \\ {\tilde{C}}_{M-1}\Phi^{-1}\\ {\tilde{C}}_{M} \end{array}\right]$$

$$\bar{D}\;≜\;\left[\begin{array}{ccccc} 0 & {\tilde{C}}_{1}\Phi^{-1} & {\tilde{C}}_{1}\Phi^{-2} & \cdots & {\tilde{C}}_{1}\Phi^{-M+1}\\ 0 & 0 & {\tilde{C}}_{2}\Phi^{-1} & \cdots & {\tilde{C}}_{2}\Phi^{-M+2}\\ \vdots & \vdots & \vdots & \ddots & \vdots \\ 0 & 0 & 0 & \cdots & {\tilde{C}}_{M-1}\Phi^{-1}\\ 0 & 0 & 0 & \cdots & 0 \end{array}\right]$$

We will apply a Kalman filter to (16). Note that

(17) $$E\{{\bar{w}}_{k}{\bar{w}}_{k}^{\prime }\}\;=\;\bar{Q}$$

where

$$\bar{Q}\;=\;\text{Diag}(Q_{d},\;Q_{d},\;\cdots ,\;Q_{d},\;Q_{d})$$

(18) $$E\{({\bar{v}}_{k}\;+\;{\bar{q}}_{k}+\;\bar{D}{\bar{w}}_{k-1})\;({\bar{v}}_{k}\;+\;{\bar{q}}_{k}\;+\;\bar{D}{\bar{w}}_{k-1})\prime \}\;=\;\bar{R}$$

where

$$\begin{array}{lll} \bar{R} & = & \text{Diag}({\tilde{R}}_{1},\;\cdots ,\;{\tilde{R}}_{M})\;+\;\bar{\Delta }\;+\;\bar{D}\bar{Q}\bar{D}\prime \\ \bar{\Delta } & = & \text{Diag}({\tilde{\Delta }}_{1},\;\cdots ,\;{\tilde{\Delta }}_{M}). \end{array}$$

(19) $$E\{\bar{B}{\bar{w}}_{k-1}\;({\bar{v}}_{k}^{\prime }\;+\;{\bar{q}}_{k}^{\prime }\;+\;(\bar{D}{\bar{w}}_{k-1})\prime )\}\;=\;\bar{M}\;=\;\bar{B}\bar{Q}\bar{D}\prime$$

It is standard to apply a Kalman filter to (16) using (17), (18) and (19): the measurement update and the time update equations are given as follows [20]:

- measurement update
  $$\begin{array}{l} K_{k}\;=\;(P_{k}^{-}\bar{C}\prime \;+\;\bar{M})\;(\bar{C}P_{k}^{-}\bar{C}\prime \;+\;\bar{R}\;+\;\bar{C}\bar{M}\\ \;\;\;\;\;\;\;\;\;\;\;\;\;\;{+\;\bar{M}\prime \bar{C}\prime )}^{-1} \end{array}$$
  $$P_{k}\;=\;P_{k}^{-}\;-\;K_{k}\;(\bar{C}P_{k}^{-}\bar{C}\prime \;+\;\bar{R}\;+\;\bar{C}\bar{M}\;+\;\bar{M}\prime \bar{C}\prime )K_{k}^{\prime }$$
- time update
  $$P_{k+1}^{-}\;=\;\bar{A}P_{k}\bar{A}\prime \;+\;\bar{B}\bar{Q}\bar{B}\prime$$

We will use *P* as an estimation performance, where *P* is the steady-state value of $P_{k}^{-}$:

$$P\;≜\;\underset{k\to \infty }{\text{lim}}\;P_{k}^{-}$$

In the steady state, we have $P_{k+1}^{-}\;=\;P_{k}^{-}\;=\;P$. Inserting this into the Kalman filter equation, we have the following Riccati equation:

(20) $$\begin{array}{c} P\;=\;\bar{A}P\bar{A}\prime \;-\;(\bar{A}P\bar{C}\prime \;+\;\bar{A}\bar{M})\\ {\left(\bar{C}P\bar{C}\prime \;+\;\bar{R}\;+\bar{C}\bar{M}\;+\;\bar{M}\prime \bar{C}\prime \right)}^{-1}\;(\bar{A}P\bar{C}\prime \;+\;\bar{A}\bar{M})\prime \\ +\;\bar{B}\bar{Q}\bar{B}\prime \end{array}$$

If the sampling period *T_i_* and the quantization bit length *l_i_* are given, the corresponding estimation performance can be computed from (20).

## 3. *T_i_* and *l_i_* Optimization

In this section, a method to select the sampling period *T_i_* and the quantization bit *l_i_* are proposed. The main trade-off is between the transmission rate and the estimation performance.

The optimization problem can be formulated as follows:

(21) $$\begin{array}{c} {\text{min}}_{T_{i},l_{i}}\;\lambda \prime \;\text{Diag}\;P\\ \text{subject to}\;\Sigma_{i=1}^{p}\;\frac{l_{i}}{T_{i}}\leq \;S_{\max} \end{array}$$

where λ ∈ *R^n^* is a weighting vector. Note that the transmission rate *S* is given by

(22) $$S\;≜\;\sum_{i=1}^{p}\frac{l_{i}}{T_{i}}$$

and *S_max_* is the transmission rate constraint. The transmission rate *S* is defined as the sum of each sensor data rate without considering packet overhead. When we apply the algorithm to a specific network, the transmission rate *S* should be modified to take the packet overhead into account.

Note that *T_i_* = *M_i_T* and *P* depends on *M*, which is the least common multiple of *M*_1_, . . ., *M_p_*. To make *M* constant, *M_i_* is assumed to satisfy

(23) $$\begin{array}{c} M_{i}\;=\;2^{m_{i}}\;(m_{i}\;\text{is an integer})\\ m_{i,min}\;\leq \;m_{i}\;\leq \;m_{i,\max} \end{array}$$

With this assumption, the least common multiple *M* of all possible combinations of *M_i_* = 2*^m_i_^* is given by

$$M\;=\;2^{\max_{i}\{m_{i,\max}\}}$$

We assume *l_i_* satisfies

(24) $$l_{i,\min}\;\leq \;l_{i}\;\leq \;l_{i,\max}$$

If the number of combinations is small, *P* can be computed for all possible combinations. For a case that the number is too large, we propose a suboptimal algorithm. The proposed algorithm is based on the following lemma.

**Lemma 1** *Let P* (*m*_1_, *l*_1_, . . ., *m_i_*, *l_i_*, . . ., *m_p_*, *l_p_*) *be the solution to (20). With the assumption (23), the following is satisfied.*

(25) $$P(m_{1},\;l_{1},\;\cdots ,\;m_{i}\;-\;1,\;l_{i},\;\cdots ,\;m_{p},\;l_{p})\;\leq \;P(m_{1},\;l_{1},\;\cdots ,\;m_{i},\;l_{i},\;\cdots ,\;m_{p},\;l_{p})$$

(26) $$P(m_{1},\;l_{1},\;\cdots ,\;m_{i},\;l_{i}\;+\;1,\;\cdots ,\;m_{p},\;l_{p})\;\leq \;P(m_{1},\;l_{1},\;\cdots ,\;m_{i},\;l_{i},\;\cdots ,\;m_{p},\;l_{p})$$

(27) $$P(m_{1},\;l_{1},\;\cdots ,\;m_{i}\;-\;1,\;l_{i}\;+\;1,\;\cdots ,\;m_{p},\;l_{p})\;\leq \;P(m_{1},\;l_{1},\;\cdots ,\;m_{i},\;l_{i},\;\cdots ,\;m_{p},\;l_{p})$$

Proof: We will prove with a simple case with *m*_1_ = 0 *m*_2_ = 0, *T* = 1, *M* = 2, and *p* = 2: note that *T*_1_ = 2^*m*_1_^*T* = *T* and *T*_2_ = 2^*m*_2_^*T* = *T. C̄* for *m*_1_ = 0 and *m*_2_ = 0 is given by

$${\bar{C}}_{m_{1}=0,m_{2}=0}\;=\;\left[\left[\begin{array}{c} C_{1}\\ C_{2}\\ C_{1}\\ C_{2} \end{array}\right]\right]$$

The subscript (*m*_1_ = 0, *m*_2_ = 0) is used to emphasize that *m*_1_ = 0 and *m*_2_ = 0. Also let *R̄*_*m*_1_=0,*m*_2_=0_ be *R̄* defined in (18) and *P*(0, *l*_1_, 0, *l*_2_) be a solution to (20) when *m*_1_ = 0 and *m*_2_ = 0.

Now consider a case with *m*_1_ = 0 and *m*_2_ = 1 Instead of computing *P*(0, *l*_1_, 1, *l*_2_) using (20) with *C̄*_*m*_1_=0,*m*_2_=1_, we can compute *P*(0, *l*_1_, 1, *l*_2_) using (20) with *m*_1_ = 0 and *m*_2_ = 0 except that *R̄*_*m*_1_=0,*m*_2_=0_ is replaced by *R̄_modified_*, which is defined by

$${\bar{R}}_{\mathrm{modified}}\;=\;{\bar{R}}_{(m_{1}=0,m_{2}=0})\;+\;\text{Diag}(0,\infty ,0,0).$$

Note that adding ∞ to the (2, 2) element of *R̄*_*m*_1_=0,*m*_2_=0_ is equivalent to ignoring the second output of *y_k_* when *k* is an integer multiple of 2. Thus *P*(0, *l*_1_, 1, *l*_2_) computed in this way is the estimation error covariance when *m*_1_ = 0 and *m*_2_ = 1. Since *R̄_modified_* ≥ *R̄*, we have from the monotonicity of the Riccati equation (see Corollary 5.2 in [21])

$$P(0,\;l_{1},\;0,\;l_{2})\;\leq \;P(0,\;l_{1},\;1,\;l_{2})$$

The general case for (25) can be proved similarly.

Proving the second inequality is more straightforward from the monotonicity of the Riccati equation [21] and from the fact

$${\bar{R}}_{(m_{1},l_{1},\cdots ,m_{i},l_{i}+1,\cdots ,m_{p},l_{p}})\;\leq \;{\bar{R}}_{\left(m_{1},l_{1},\cdots ,m_{i},l_{i},\cdots ,m_{p},l_{p}\right)}$$

The third inequality (27) is just a combination of (25) and (26):

$$\begin{array}{c} P(m_{1},\;l_{1},\;\cdots ,\;m_{i}\;-1,\;l_{i}\;+\;1,\;\cdots ,\;m_{p},\;l_{p})\;\leq \;P(m_{1},\;l_{1},\;\cdots ,\;m_{i},\;l_{i}\;+\;1,\;\cdots ,\;m_{p},\;l_{p})\\ \leq \;P(m_{1},\;l_{1},\;\cdots ,\;m_{i},\;l_{i},\;\cdots ,\;m_{p},\;l_{p}) \end{array}$$

To explain Lemma 1, we consider a simple example with the following parameters:

(28) $$\begin{array}{c} p=2\\ m_{1,\min}\;=\;m_{2,\min}\;=\;0,\;m_{1,\max}\;=\;m_{2,\max}\;=\;4\\ l_{1,\min}\;=\;l_{2,\min}\;=\;9,\;l_{1,\max}\;=\;l_{2,\max}\;=\;16 \end{array}$$

**Figure 1.**
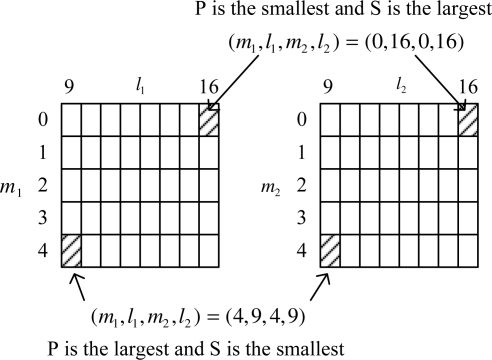
*P* (estimation error covariance) and *S* (transmission rate) relationship according to combinations.

There are 5 × 8 × 5 × 8 = 1, 600 possible combinations (see Figure 1). Using the result in Lemma 1, we know that *P* is the smallest when (*m*_1_, *l*_1_, *m*_2_, *l*_2_) = (0, 16, 0, 16) and the largest when (*m*_1_, *l*_1_, *m*_2_, *l*_2_) = (4, 9, 4, 9). On the other hand, the transmission rate *S* is the largest when (*m*_1_, *l*_1_, *m*_2_, *l*_2_) = (0, 16, 0, 16) and the smallest when (*m*_1_, *l*_1_, *m*_2_, *l*_2_) = (4, 9, 4, 9). Note that *m_i_* *−* 1 and *l_i_* + 1 in Lemma 1 corresponds to the upper and right combination of (*m_i_*, *l_i_*), respectively. Thus as we move the combination from the left-bottom corner toward the right-top corner, λ′*P* becomes smaller while the transmission rate increases.

In the proposed algorithm, we start from the left-bottom corner combination and move the combination toward the right-top corner combination while the transmission constrained is satisfied. The proposed algorithm is stated in pseudo-codes.

(*m_i_*, *l_i_*) = (*m_i,max_*, *l_i,min_*), *i* = 1, . . ., *p*
compute λ′*P* and *S*
(*P* denotes *P*(*m*_1_, *l*_1_, . . ., *m_p_*, *l_p_*) and *S* is from (22))
while (*S < S_max_*)
  L = { }
  for *i* = 1:*p*
    if (*m_i_* > *m_i,min_*)
      *L* = *L* ∪ {(*m*_1_, *l*_1_, . . ., *m_i_* − 1, *l_i_*, . . ., *m_p_*, *l_p_*)}
    if (*l_i_* < *l_i,max_*)
      *L* = *L* ∪ {(*m*_1_, *l*_1_, . . ., *m_i_*, *l_i_* + 1, . . ., *m_p_*, *l_p_*)}
  end
  for every element of *L*, compute *G̃_j_*
  $${\tilde{G}}_{j}\;=\;\frac{\lambda \prime P\;-\;\lambda \prime {\tilde{P}}_{j}}{{\tilde{S}}_{j}\;-\;S}$$
    where *P̃_j_* = *P* for the *j*-th element of *L*
    and *S̃_j_* = *S* for the *j*-th element of *L*
  (*m_i,old_*, *l_i,old_*) = (m*_i_*, *l_i_*), *i* = 1, . . ., *p*
  Find the maximum of *G_j_* and choose the
    corresponding combination as (*m_i_*, *l_i_*)
  compute λ′*P* and *S*
end
Choose the combination (*m_i,old_*, *l_i,old_*)

Note that *G̃_j_* represents the estimation performance improvement per transmission rate increase. For each given (*m_i_*, *l_i_*), we choose (*m_i_* − 1, *l_i_*) and (*m_i_*, *l_i_* + 1) for the next combination candidates. There are at most 2*^p^* combinations. Among the combinations, we find a combination of which *G̃_j_* is the largest. This process is continued until the current transmission rate exceeds *S_max_*.

The number of combinations tested in the proposed algorithm is small compared with the brute force search. For example, the proposed algorithm starts with the top-right-most combination (*m*_1_, *l*_1_, *m*_2_, *l*_2_) = (0, 16, 0, 16) and moves toward bottom-left-most combination (*m*_1_, *l*_1_, *m*_2_, *l*_2_) = (4, 9, 4, 9) at each step until *S* ≤ *S_max_* is no longer satisfied. Unfortunately, there is no guarantee that the solution found by the proposed method is near the optimal solution. In Section 4., it is shown, however, through a numerical example that the gap between the suboptimal and optimal solution is not large.

We note that the optimization algorithm is applied once when the networked system is designed. Once the sampling period *T_i_* and quantization length *l_i_* are determined, they are programmed in each sensor node. Thus no additional computation is needed in the sensor node.

## 4. Numerical Example

In this section, the proposed method is verified for the one dimensional attitude estimation problem. The state is defined by

$$x(t)\;=\;\left[\begin{array}{c} \theta \\ \dot{\theta }\\ \ddot{\theta } \end{array}\right]$$

where *θ* is the attitude we want to estimate. An accelerometer-based inclinometer and a gyroscope are used as sensors. The system model is given by

$$\begin{array}{l} A\;=\;\left[\begin{array}{ccc} 0 & 1 & 0\\ 0 & 0 & 1\\ 0 & 0 & 0 \end{array}\right],\;C=\;\left[\begin{array}{lll} 1 & 0 & 0\\ 0 & 1 & 0 \end{array}\right]\\ Q\;=\;\left[\begin{array}{ccc} 0 & 0 & 0\\ 0 & 0 & 0\\ 0 & 0 & 0.2 \end{array}\right],\;R\;=\;\left[\begin{array}{cc} 0.0056 & 0\\ 0 & 0.003 \end{array}\right] \end{array}$$

The values given in (28), *y_max_*_,1_ = 3.1416, *y_max_*_,2_ = 2.6180 and *T* = 1 are used.

The optimization problem (21) with *S_max_* = 500 and λ = [1 0 0]′ is considered. λ is a natural choice since we want to estimate *θ*.

First the optimization problem is solved by a brute force search: all possible combinations are examined. The optimal solution is given by

$$\left(m_{1},\;l_{1},\;m_{2},\;l_{2})\;=\;(2,\;10,\;2,\;10\right)$$

and *S* and λ*P* at the combination are

S = 500, λP = 0.00259.

Secondly the proposed suboptimal algorithm is used, where the solution is given by

$$\left(m_{1},\;l_{1},\;m_{2},\;l_{2})\;=\;(2,\;9,\;2,\;9\right)$$

and *S* and λ*P* at the combination are

S = 450, λP = 0.00260.

The proposed method was able to find a nearly optimal solution with less computation time. In the brute force search, 479 combinations are tested while 21 combinations are tested in the proposed algorithm.

To test whether the proposed λ*P* is a good indicator of the estimation performance, the data is generated with Matlab and tested with a Kalman filter. The estimation performance is evaluated with the following:

$$P_{\mathrm{experiment}}\;=\;\frac{1}{N}\;\sum_{k,1}^{N}\theta_{\mathrm{error},k}^{2}$$

where *N* is the number of data and *θ_error,k_* = *θ − θ̂*. Note that *θ̂* is computed by [1 0 0] *◯_k_*. We computed *P_experiment_* for all possible combinations and the minimizing combination is given by

$$\left(m_{1},\;l_{1},\;m_{2},\;l_{2})\;=\;(2,\;9,\;2,\;10\right)$$

and *S* and *P_experiment_* at the combination is

$$S=\;475,\;P_{\mathrm{experiment}}\;=\;0.00159$$

**Figure 2.**
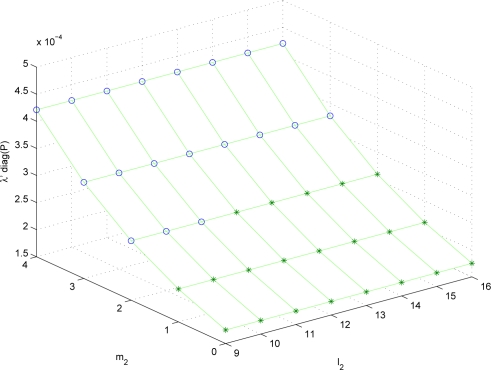
λ*P* plot with (*m*_1_, *l*_1_) = (2, 9) (o: *S* ≤ *S_max_*, *: *S > S_max_*)

**Figure 3.**
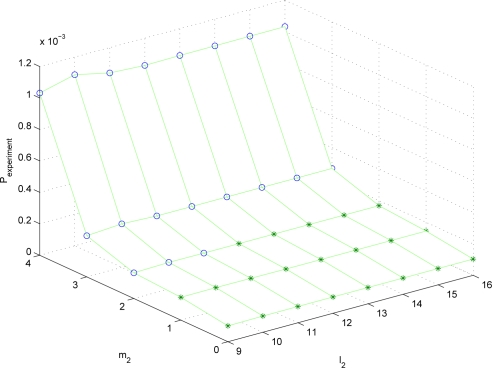
*P_experiment_* plot with (*m*_1_, *l*_1_) = (2, 9) (o: *S* ≤ *S_max_*, *: *S > S_max_*)

It can be seen that the optimal solution predicted by λ*P* nearly coincides with the real optimal solution. To see how similar λ*P* and *P_experiment_* are, λ*P* and *P_experiment_* are plotted for different (*m*_1_, *l*_1_, *m*_2_, *l*_2_) combinations. Since the parameter space is four dimensional, it is not easy to visualize the result. Thus we fix (*m*_1_, *l*_1_) = (2, 9) and plot λ*P* and *P_experiment_* for (*m*_2_, *l*_2_) combinations in Figures 2 and 3. The data marked with “o” satisfies *S* ≤ *S_max_* and the data marked with “*” does not satisfy *S* ≤ *S_max_*.

It can be seen that the trend of λ*P* is almost similar to that of *P_experiment_*. Thus λ*P* can be used to predict the estimation performance given the sampling periods and the quantization bit lengths.

**Figure 4.**
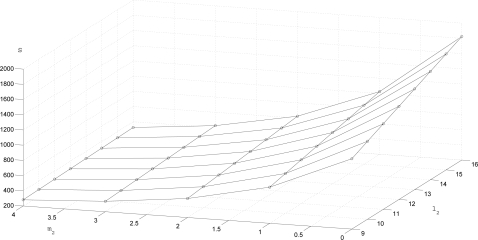
*S* plot with (*m*_1_, *l*_1_) = (2, 9)

The transmission rate *S* is given in Figure 4. To see the trade-off between *S* and the estimation performance, *S* and λ*P* are given for three (*m*_1_, *l*_1_, *m*_2_, *l*_2_) combinations in Table 2. We can see that when *S* decreases (that is, if we transmit less data), λ*P* tends to increase (that is, the estimation performance degrades).

Finally, to test the efficiency of the proposed algorithm, we applied the proposed algorithm to 100 random models, where *A* is randomly generated and the same *C* as in the previous simulation is used. In the brute force method, 479 combinations are tested as in the previous simulation since the same setting is used. In the proposed algorithm, the number of combinations tested is between 13 and 21. That is, the number of combinations tested in the worst case is 21. Thus we can see the convergence rate is relatively fast. To see the accuracy of the proposed algorithm, the following value is computed:

$$\text{Accuracy}\;=\;\lambda \frac{100\;\times \;(P_{\mathrm{proposed}}\;-\;P_{\mathrm{optimal}})}{P_{\mathrm{optimal}}}$$

where *P_optimal_* is computed using the brute force method. In the 100 trials, the worst case accuracy was 7.26% while the average value is 1.73%. Thus we believe the proposed method can find near optimal value while avoiding the large computation.

## 5. Conclusions

In this paper, attitude estimation over a network with a transmission rate constraint is considered. The transmission rate depends on the sampling periods and the quantization bit lengths. Basically the problem is trade-off between the estimation performance and the transmission rate, where the parameters are the sampling period and the quantization bit length.

First, how the sampling period and the quantization bit length affect the estimation performance is investigated. To do this, we introduced an augmented system and defined the estimation performance *P*. Secondly, the trade-off problem is formulated as an optimization problem and a suboptimal algorithm is provided. Through numerical examples, we showed that the defined estimation performance matches the real estimation performance in the sense that graphs of *P* and the real estimation performance are similar. We also showed the proposed algorithm could find a reasonably good solution.

While defining *P*, we made assumption (23), which makes the derivation of *P* easier but not essential. Removing that assumption and obtaining a general result is a future work. Also to test an algorithm using a real network is a future work.

## Figures and Tables

**Table 1.** *ỹ_k_* and *C̃_k_* example (*M*_1_ = 1 and *M*_2_ = 2)

*k*	1	2	3	4
*ỹ_k_*	[*y_k,_*_1_]	$\left[\begin{array}{c} y_{k,1}\\ y_{k,2} \end{array}\right]$	[*y_k,_*_1_]	$\left[\begin{array}{c} y_{k,1}\\ y_{k,2} \end{array}\right]$
*C̃_k_*	[*C*_1_]	$\left[\begin{array}{c} C_{1}\\ C_{2} \end{array}\right]$	[*C*_1_]	$\left[\begin{array}{c} C_{1}\\ C_{2} \end{array}\right]$

**Table 2.** *S* and λ*P* comparison

*m*_1_	*l*_1_	*m*_2_	*l*_2_	*S*	λ*P*
0	16	0	16	3200	0.000080
2	12	2	12	600	0.000165
4	9	4	9	112.5	0.001282
